# TGF*β* signaling related genes are involved in hormonal mediation during termite soldier differentiation

**DOI:** 10.1371/journal.pgen.1007338

**Published:** 2018-04-11

**Authors:** Yudai Masuoka, Hajime Yaguchi, Kouhei Toga, Shuji Shigenobu, Kiyoto Maekawa

**Affiliations:** 1 Graduate School of Science and Engineering, University of Toyama, Toyama, Japan; 2 Institute of Agrobiological Sciences, National Agriculture and Food Research Organization, Tsukuba, Ibaraki, Japan; 3 Tropical Biosphere Research Center, University of the Ryukyus, Okinawa, Japan; 4 Department of Biosciences, College of Humanities and Sciences, Nihon University, Tokyo, Japan; 5 Functional Genomics Facility, National Institute for Basic Biology, Okazaki, Japan; New York University, UNITED STATES

## Abstract

A working knowledge of the proximate factors intrinsic to sterile caste differentiation is necessary to understand the evolution of eusocial insects. Genomic and transcriptomic analyses in social hymenopteran insects have resulted in the hypothesis that sterile castes are generated by the novel function of co-opted or recruited universal gene networks found in solitary ancestors. However, transcriptome analysis during caste differentiation has not been tested in termites, and evolutionary processes associated with acquiring the caste are still unknown. Termites possess the soldier caste, which is regarded as the first acquired permanently sterile caste in the taxon. In this study, we performed a comparative transcriptome analysis in termite heads during 3 molting processes, i.e., worker, presoldier and soldier molts, under natural conditions in an incipient colony of the damp-wood termite *Zootermopsis nevadensis*. Although similar expression patterns were observed during each molting process, more than 50 genes were shown to be highly expressed before the presoldier (intermediate stage of soldier) molt. We then performed RNA interference (RNAi) of the candidate 13 genes, including transcription factors and uncharacterized protein genes, during presoldier differentiation induced by juvenile hormone (JH) analog treatment. Presoldiers induced after RNAi of two genes related to TGF*β* (Transforming growth factor beta) signaling were extremely unusual and possessed soldier-like phenotypes. These individuals also displayed aggressive behaviors similar to natural soldiers when confronted with *Formica* ants as hypothetical enemies. These presoldiers never molted into the next instar, presumably due to the decreased expression levels of the molting hormone (20-hydroxyecdysone; 20E) signaling genes. These results suggest that TGF*β* signaling was acquired for the novel function of regulating between JH and 20E signaling during soldier differentiation in termites.

## Author summary

The acquisition of a sterile caste is a key step in animal eusocial evolution. The soldier is the first acquired permanently sterile caste in termites, which are distantly related to hymenopteran eusocial insects (bees, ants and wasps). However, the evolutionary background to acquiring the soldier caste is still largely unclear. Here we performed transcriptome analysis of heads during worker and soldier caste differentiation under natural conditions in *Zootermopsis nevadensis*, for which the whole-genome sequence is available. Soldiers differentiate from workers via a double molting processes, and the intermediate stage is called a presoldier. Based on the comparison among molting stages, some presoldier-specific highly expressed genes, including transcription factors and several uncharacterized protein genes, were identified. RNAi of two genes presumably involved in the TGF*β* signaling resulted in the formation of presoldiers possessing soldier-like phenotypes (e.g. well-tunned cuticle) without further molting into soldiers. Expression levels of 20E signaling genes were negatively affected by the RNAi treatment of these genes. This study provides a novel insight into the hormonal control for termite caste differentiation.

## Introduction

The complex society of eusocial insects includes sterile castes with phenotypes specialized for individual social tasks. To clarify the mechanisms associated with acquiring sterile castes is a fundamental goal in the evolutionary biology of eusocial insects. In hymenopteran species (bees, ants and wasps), many sociogenomic studies have been conducted in the last decade (reviewed in [1,2]) based on a vast quantity of genomic and transcriptomic information. These studies suggested that a co-option of the universal gene network in the solitary ancestor was involved in the evolution of sterile castes [3–7]. However, in termites, which are distantly related to hymenopteran social insects, there is still no evidence to support the importance of a universal gene network for the evolution of sterile castes, largely because genetic profiles during caste differentiation have not been clarified. One of the most important species for addressing this issue is the damp-wood termite *Zootermopsis nevadensis*, because genomic and transcriptomic information is available [8], gene function analysis has been successfully applied [9–11], and sterile caste differentiation can be observed under natural conditions [9,12] as well as under artificial hormone treatment [10].

In contrast to hymenopteran eusocial insects, in termites the soldier is regarded as the first acquired permanently sterile caste [13]. Soldiers exhibit unique species-specific morphology, which is formed through developmental processes that include double molts (worker—presoldier and presoldier—soldier). Since presoldiers cannot revert to workers, this stage is necessary for soldier-specific morphological changes (e.g. cuticular formation [10]). These processes are not observed in other insects, and are accompanied by extraordinary phenotypic modifications similar to metamorphosis in holometabolous insects [14]. Soldier differentiation is regulated by juvenile hormone (JH), and high JH titer levels in workers trigger soldier differentiation [15]. Indeed, it was reported that JH receptor gene (*Methoprene tolerant*: *Met*) expression was involved in soldier-specific morphogenesis during soldier molts in *Z*. *nevadensis* [9]. Moreover, the molting hormone (20-hydroxyecdysone; 20E) is also crucial for soldier differentiation. Ecdysone receptor gene (*EcR*) expression was also involved in presoldier molt and soldier-specific cuticular pigmentation in *Z*. *nevadensis* [11]. However, regulatory mechanisms of a crosstalk between JH and 20E to generate highly specialized phenotypes are still a mystery. One possibility is that it is mediated by the universal gene network commonly possessed by other hemimetabolous insects, such as wnt and TGF*β* (Transforming growth factor beta) signalings, both of which have multiple roles for embryogenesis and hormonal regulation. Especially, TGF*β* signaling should be a focus, because it was reported as a regulator of JH synthesis in the cricket, *Gryllus bimaculatus* [16]. Moreover, TGF*β* signaling was involved in the neuronal development and metamorphosis in *Drosophila melanogaster* and the German cockroach, *Blattella germanica* [17,18]. To clarify the intrinsic mechanism of soldier differentiation mediated by hormonal changes, comparative transcriptome analysis of the head during each molting process under natural conditions will be most effective, because both JH and ecdysone biosynthetic organs (corpora allata and molt gland, respectively) exist in the insect head.

In this study, transcriptome analysis (RNA-seq) was performed in *Z*. *nevadensis* to detect an unknown regulatory factor involved in crosstalk between JH and 20E during soldier differentiation. In an incipient colony of this species, the oldest 3rd instar larva (No. 1 larva) molts into a presoldier, whereas the next-oldest 3rd instar larva (No. 2 larva) molts into the next instar (4th instar larva). Gut-purged individuals (those with the elimination of gut contents before the molt) are always observed 3 days prior to molt. Therefore, the time frame of worker, presoldier and soldier molts can be determined by records of the day of molt or gut-purge [9,12]. Note that because individuals developed beyond the 2nd instar function as workers, they are referred to as workers in this study. Based on the RNA-seq analysis, some important candidate genes were identified, all of which showed increased expressions in the head before the presoldier molt. The function of these genes and relationships with JH and/or 20E during soldier differentiation were confirmed by RNA interference (RNAi) and qPCR analysis. Based on the results obtained, we discuss how termite soldiers are differentiated with marked morphological modification via the double molting processes.

## Results

### Detection of highly expressed genes in the head before the presoldier molt

**Fig 1:**
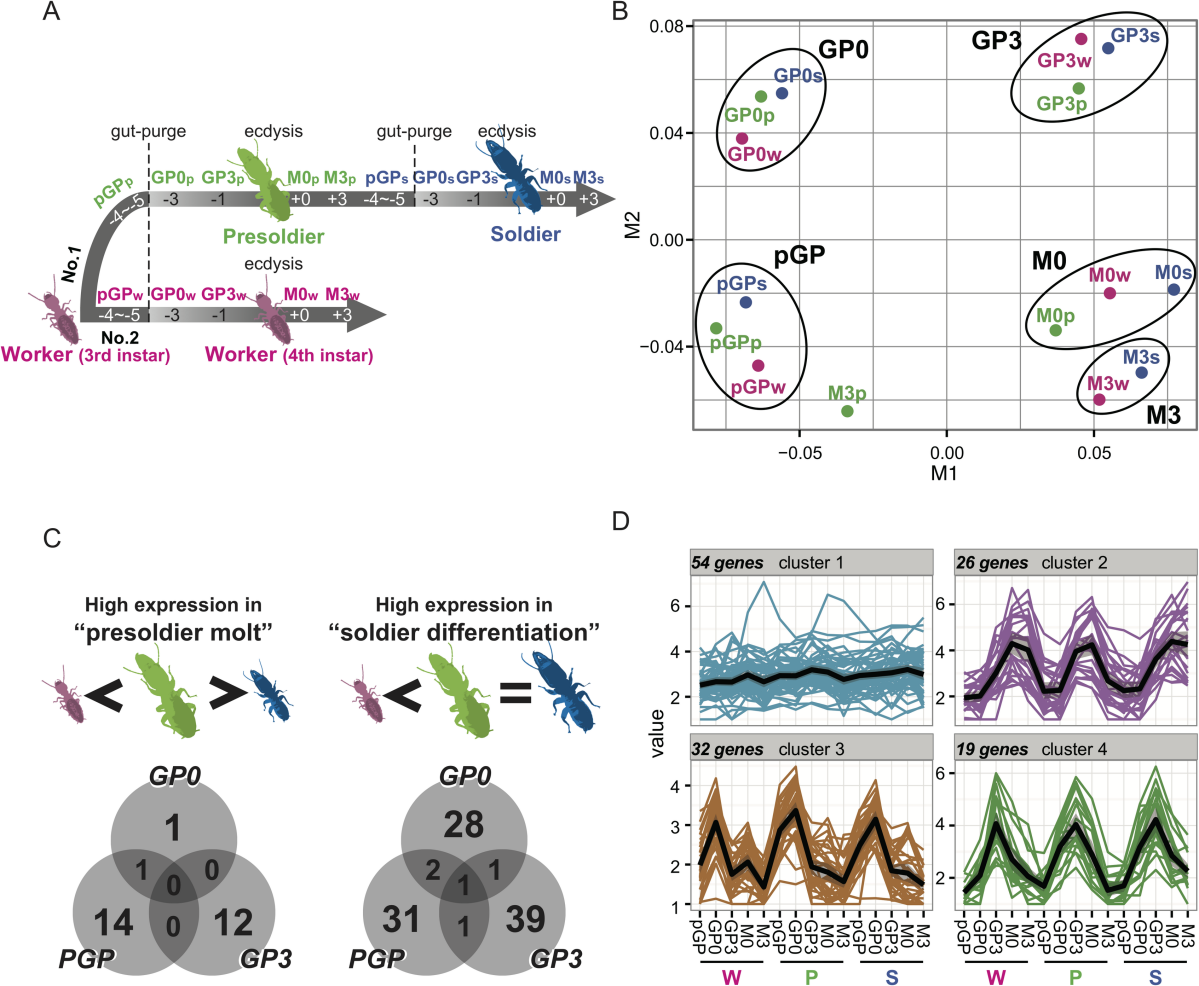
Sampling points for RNA-seq analysis and gene expression profiles during each molting process. (A) Caste differentiation observed in an incipient colony of *Zootermopsis nevadensis*. The oldest 3rd instar larva (No. 1 larva) molts into a presoldier, whereas the next-oldest 3rd instar larva (No. 2 larva) molts into the next instar (4th instar larva). Gut-purged individuals are always observed 3 days before the molts. Individuals were sampled at the following 5 developmental stages during each molt; pre-gut-purging (pGP), 0 days after gut-purging (GP0), 3 days after gut-purging (GP3), 0 days after the molt (M0) and 3 days after the molt (M3). (B) MDS plots of 14,204 genes detected by the RNA-seq data from the head during 3 molts in *Zootermopsis nevadensis*. (C) Numbers of caste-specific highly expressed genes among 3 timings in each molt (pGP, GP0, GP3). (D) Clusters of genes with highly expression levels in the head before the presoldier molt. Cluster 1 was composed of 54 genes showing no clear expression patterns with the molting events. Cluster 2 was composed of 26 genes with highly expression levels after the molt (M0) in each molting event. Cluster 3 and 4 were composed of 32 and 19 genes with highly expression levels before the molt (GP0 and GP3, respectively). The solid black line in each panel indicates the average expression pattern. The similarity was calculated based on the Jensen-Shannon distances using the RPKM (Reads Per Kilo base of exon model per Million mapped reads) values.

The expression levels of large numbers of genes (14,204/15,876 genes, 89.5% of all genes described in the model OGSv2.2; [8]) were observed by the present RNA-seq data from 3 molting processes (Fig 1A). MDS (multi-dimensional scaling) of all expression patterns were similar to one another during the 3 molting processes (Fig 1B). Namely, similar expression patterns were observed in the same developmental stage (pGP, GP0, GP3, M0 or M3), not in each molting process. Note that the expression pattern of the M3 library (three days after the molt) in the presoldier molt (M3p; Fig 1A) was similar to that of the pGP library (pre-gut-purging). This was due to the nearly equal sampling points of the former and the pGP library in the soldier molt (pGPs; Fig 1A). To narrow down the crucial genes of presoldier development, significantly highly expressed genes were investigated by a comparison with the same developmental stage (pGP, GP0 and GP3). Highly expressed genes in the presoldier molt compared with those in worker and soldier molts (i.e. pGPp > pGPw and pGPp > pGPs, GP0p > GP0w and GP0p > GP0s, GP3p > GP3w and GP3p > GP3s) were 28 genes in total (Fig 1C, S1 Table). Moreover, 103 genes were more highly expressed in the presoldier molt than those in the worker molt and equally expressed compared with soldier molt (i.e. pGPp > pGPw and pGPp = pGPs, GP0p > GP0w and GP0p = GP0s, GP3p > GP3w and GP3p = GP3s; Fig 1C, S1 Table). Consequently, a total of 131 genes were identified, but these numbers were below 1% of all genes observed in this study. Expression patterns of these genes were divided into 4 clusters by cummeRbund (Fig 1D). Expression patterns of 77 out of 131 genes were shown to be related to the molting process (Cluster 2–4). On the other hand, expression patterns of the remaining 54 genes were not related to the molting process, and might be different during each molt (Cluster 1). Based on the annotation of these 54 genes by the FlyBase and nr database in NCBI, 3 transcription factors, 1 JH binding protein and some uncharacterized protein genes were observed (S2 Table). The following functional analysis was performed in these target genes.

### Functional analysis of candidate genes during the presoldier molt

**Fig 2:**
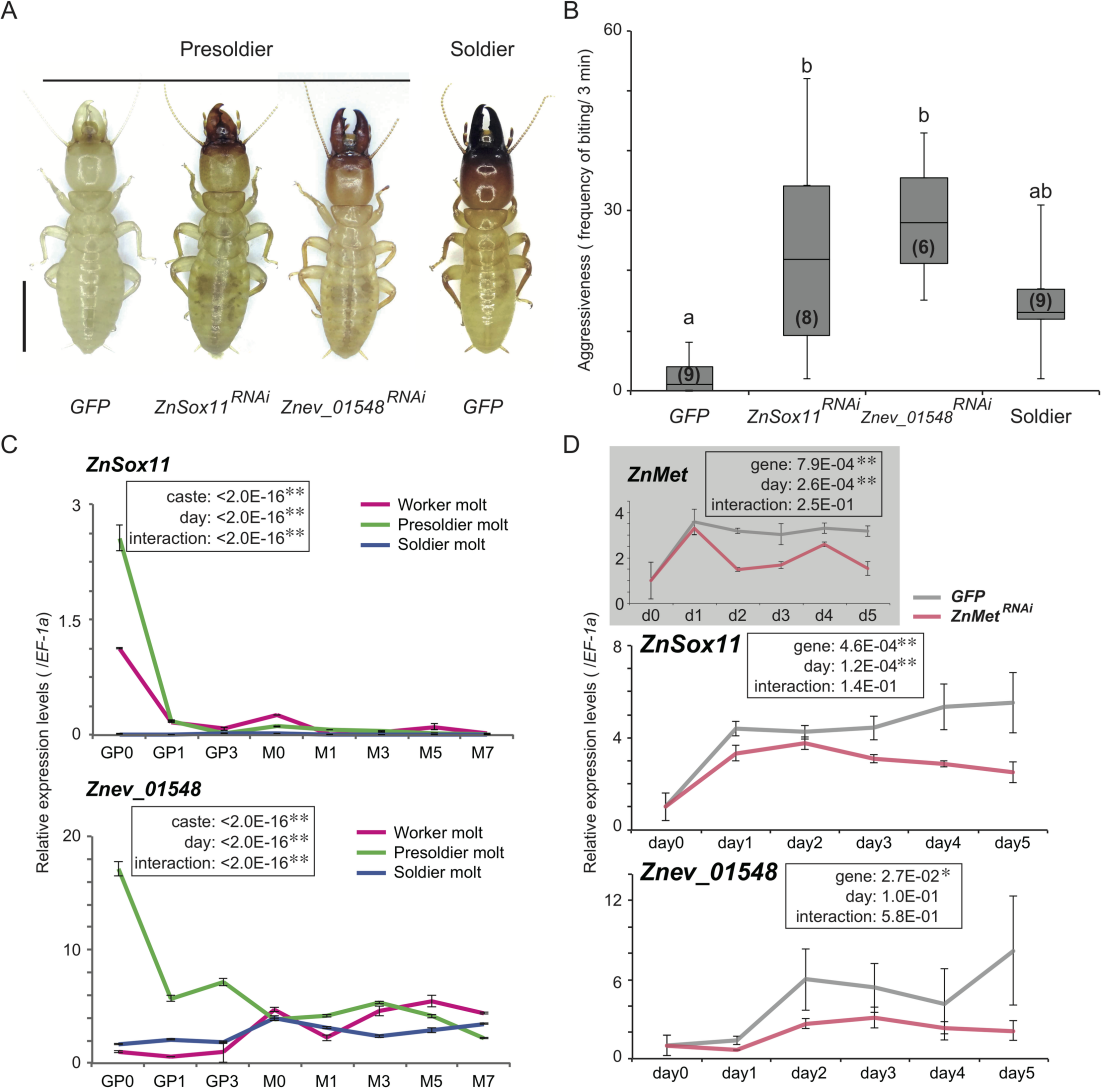
RNAi effects and expression patterns of *ZnSox11* and *Znev_01548*. (A) Phenotypes of *GFP*, *ZnSox11* and *Znev_01548* dsRNA injected presoldiers. Each dsRNA was injected into the side of the thorax 24 hours after the JH analog (JHA) application. The emerged presoldiers were photographed 7 days after the molt. Approximately half of the *GFP* dsRNA injected presoldiers (14 out of 26, 53.8%) molted into soldiers with normal phenotypes (right). Scale bar indicates 5 mm. (B) Aggression levels of *GFP*, *ZnSox11* and *Znev_01548* dsRNA injected presoldiers (n = 6–9). The levels of natural soldiers are also shown on the right (n = 9). Numbers of individuals examined are shown in parentheses. The boxes and whiskers mean median, quartiles and range. Different letters over the bars indicate significant differences in each category (Kruskal-Wallis test: P = 6.35E-04, Steel-Dwass test: P < 0.05). (C) Expression patterns of *ZnSox11* and *Znev_01548* in the head provided by the qPCR analysis during each molting process in an incipient colony. Relative expression levels (mean ± S.E., biological triplicates) were calibrated by the expression levels in workers (GP0) as 1.0. The statistical results of three-way ANOVA are described in each box (**P < 0.01). The data is consistent with the use of parametric statistics by the Browne-Forsythe test (*ZnSox11*: P = 6.37E-01 (worker), 5.08E-02 (presoldier), 7.75E-01 (soldier); *Znev_01548*: P = 6.77E-01 (worker), 7.45E-01 (presoldier), 9.08E-01 (soldier)) prior to the use of the ANOVAs. (D) Expression patterns of *ZnSox11* and *Znev_01548* under the *ZnMet* RNAi treatment. Gray and red lines indicate the results under the *GFP* and *ZnMet* RNAi treatments, respectively. Relative expression levels (mean ± S.E., biological triplicates) were calibrated by the expression level of intact worker (day 0) as 1.0. The statistical results of two-way ANOVA are described in each box (*P < 0.05, **P < 0.01). The data is consistent with the use of parametric statistics by the Browne-Forsythe test (*ZnMet*: P = 7.91E-01 (*GFP*), 5.90E-01 (*ZnMet* RNAi); *ZnSox11*: P = 4.99E-01 (*GFP*), 8.43E-01(*ZnMet* RNAi); *Znev_01548*: P = 6.46E-01 (*GFP*), 6.25E-01 (*ZnMet* RNAi)) prior to the use of the ANOVAs.

For the screening of genes crucial to soldier differentiation, RNAi treatments of some genes were performed during presoldier molt. A total of 13 candidate genes, including transcription factors (*Znev_04641*, *Znev_05644* and *Znev_11299*), JH binding protein (*Znev_03428*) and function-unknown hypothetical genes in *D*. *melanogaster* (total 9), were selected for the following functional analysis. There is a possibility that these genes have a crucial role for soldier differentiation, because they may have an important role in JH-dependent gene expression changes. Due to the limited numbers of soldier-destined individuals in the incipient colonies (basically only one individual in each colony; [12]), functional analysis was conducted using the JH analog (JHA)-induced presoldier differentiation experiments. The knockdown of 8 out of 13 focal genes did not have any effects on the individuals, and normal presoldiers were induced by JHA treatment (S3 Table). RNAi of three genes (*Znev_05644*, *Znev_15631* and *Znev_16430*) had a lethal effect in JHA treated workers (S3 Table). On the other hand, RNAi of *Znev_04641* (*transcription factor SOX-11-like isoform X3*; hereafter called *ZnSox11*) and *Znev_01548* (uncharacterized protein gene) resulted in noteworthy phenotypes after the presoldier molt induced by the JHA treatment compared with the *GFP* controls (Fig 2A). Namely, both RNAi-treated individuals possessed soldier-like phenotypes with well-tanned cuticle despite being the 1st molting stage from workers (in this case, the 7th instar larvae). Moreover, behavioral analysis showed that biting frequency in response to the presence of the ant *F*. *japonica* was significantly higher than that of the *GFP* controls and similar to that of natural soldiers (Fig 2B, S4 Table). In the *GFP* controls, the 2nd (i.e., induced soldier) molts were observed within 30 days after the 1st molt (molting rates: 53.8%, n = 14/26). However, the 2nd molts were never observed in both *ZnSox11* and *Znev_01548* RNAi-treated individuals within the same periods (n = 0/25 and 0/27, respectively). The color nature values (HSB color model) of head capsules of both *ZnSox11* and *Znev_01548* RNAi-treated individuals were intermediate between presoldiers (= *GFP* RNAi-treated individuals) and soldiers (S1 Fig, S5 Table). The double knockdown of *ZnSox11* and *Znev_01548* caused phenotypes similar to single knockdown of each gene (S1 Fig, S5 Table). High expressions of both genes before the presoldier molt in an incipient colony were confirmed by the real-time qPCR using head samples (3 biological replicates) (Fig 2C, S6 Table). Moreover, expression levels of both genes in the whole body were inhibited by RNAi treatment of JH receptor gene, *Methoprene tolerant* (*Met*), during artificial presoldier molting processes (Fig 2D, S6 Table). Based on the blast search, we observed that *Znev_01548* contained TGF*β* (Transforming growth factor beta) propeptide domain (E-value = 1.71e-4: NCBI, S2 Fig).

### Expression analysis under RNAi treatment of *ZnSox11* and *Znev_01548*

**Fig 3:**
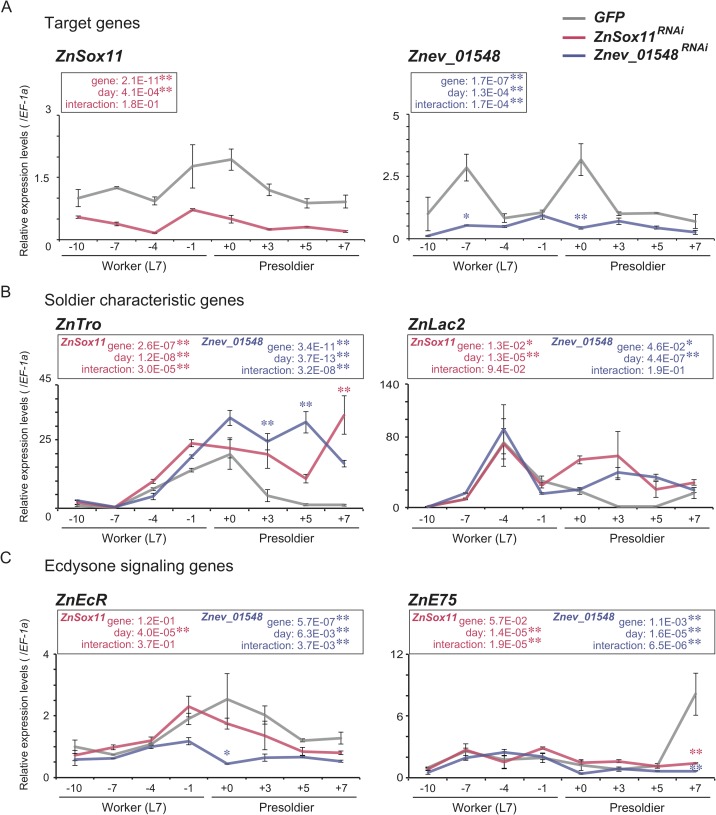
Gene expression during presoldier molt induced by JHA treatment under *ZnSox11* and *Znev_01548* RNAi treatment. **(**A) Expression patterns in the whole body of *ZnSox11* and *Znev_01548*, (B) soldier characteristic genes and (C) 20E signaling genes. Gray, red and blue lines indicate the results under the *GFP*, *ZnSox11* and *Znev_01548* RNAi treatments, respectively. Relative expression levels (mean ± S.E., biological triplicates) were calibrated by the expression level of *GFP* dsRNA injected workers (-10) as 1.0. The statistical results of two-way ANOVA are described in each box (*P < 0.05, **P < 0.01). Asterisks over the bars indicate significant differences in each gene compared with the *GFP* control (Scheffe’s F test: *P < 0.05, **P < 0.01). The data is consistent with the use of parametric statistics by the Browne-Forsythe test (*ZnSox11*: P = 5.08E-01 (*GFP*), 5.73E-01 (*ZnSox11* RNAi); *Znev_01548*: P = 7.39E-01 (*GFP*), 7.94E-01 (*Znev_01548* RNAi); *ZnTro*: P = 9.78E-02 (*GFP*), 5.17E-01 (*ZnSox11* RNAi), 3.86E-01 (*Znev_01548* RNAi); *ZnLac2*: P = 1.40E-01 (*GFP*), 6.12E-01 (*ZnSox11* RNAi), 2.86E-01 (*Znev_01548* RNAi); *ZnEcR*: P = 5.82E-01 (*GFP*), 8.62E-01 (*ZnSox11* RNAi), 4.42E-01 (*Znev_01548* RNAi); *ZnE75*: P = 2.97E-01 (*GFP*), 4.90E-01 (*ZnSox11* RNAi), 7.91E-01 (*Znev_01548* RNAi)) prior to the use of the ANOVAs.

RNAi of *ZnSox11* and *Znev_01548* significantly inhibited the same gene expression levels in heads of JHA-treated individuals compared with the *GFP* control (Fig 3A, S6 Table). The expression levels of soldier characteristic genes, including *ZnTro* and *ZnLac2*, were activated by RNAi treatment of two genes, especially after the molt, compared with the *GFP* control (Fig 3B, S6 Table). Furthermore, expression of 20E signaling genes after the molt was also influenced by the RNAi treatments (Fig 3C, S6 Table). Expression levels of *ZnEcR* in the head just after the JHA-induced presoldier molt were significantly inhibited by *Znev_01548* RNAi compared with the *GFP* control. Expression levels of *ZnE75* were also significantly reduced by RNAi of both genes (Fig 3C, S6 Table). On the other hand, expressions levels of ecdysone synthetic genes were not decreased by either RNAi treatment (S3 Fig, S6 Table).

## Discussion

### Gene expression pattern during soldier differentiation

RNA-seq analysis demonstrated that expression patterns of all mapped genes were essentially similar in three molting processes (worker, presoldier and soldier molts) under natural conditions. These results suggest that soldier differentiation is not related to the large gene expression changes exhibited during postembryonic larval molts. It has been proposed that co-option of a few genes in the influential gene network drives evolution of novel traits, including the sterile caste of hymenopteran social insects [5,6]. Similar expression patterns among each of the molts observed here may support that the co-option of a few key genes is also involved in the formation of termite soldiers. Comparative transcriptome analysis before each molt (pGP, GP0 or GP3) provided 131 genes as significantly highly expressed in the head during presoldier differentiation. However, many genes (77 genes, 58.8%) exhibited similar expression patterns through each molting process regardless of their phenotypic differences (Fig 1D). There is a possibility that the remaining genes (54 genes, 41.2%) possess important functions for soldier differentiation, including a specific morphogenesis and double molting system.

### Role of TGF*β* signaling related genes during soldier differentiation

**Fig 4:**
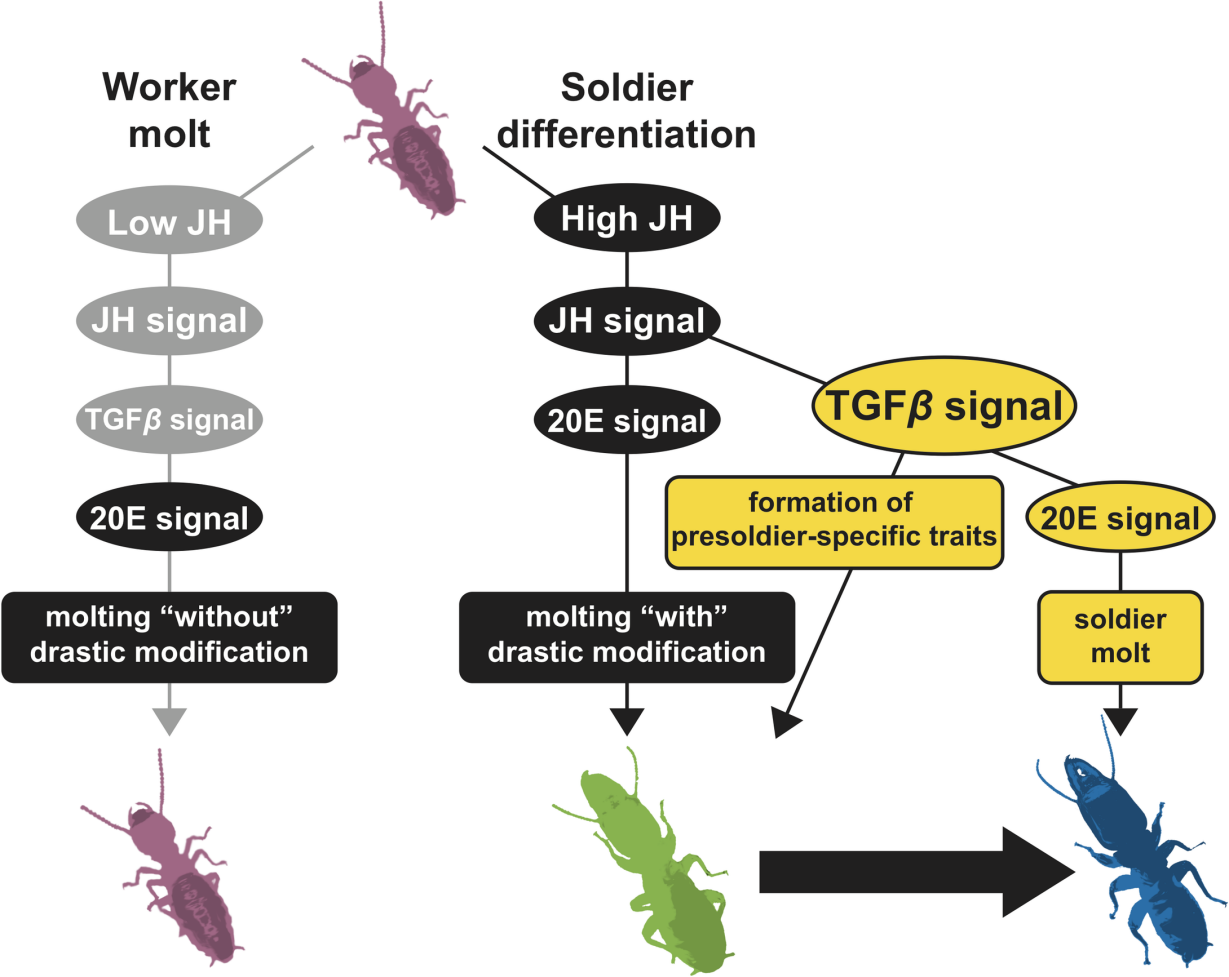
A schematic model on the role of TGF*β* signaling for soldier differentiation. When JH titer is increased in larvae, TGF*β* signaling may be involved in the formation of presoldier-specific traits (e.g. soft cuticle, low aggression) and the regulation of soldier molt via 20E signaling.

Within 54 genes identified (S2 Table), we especially focused on the transcription factors (*Znev_04641*, *Znev_05644* and *Znev_11299*), JH binding protein (*Znev_03428*) and function-unknown hypothetical genes (total 9). Functional analysis using the artificial presoldier induction method showed that similar phenotypic effects were caused by *ZnSox11* and *Znev_01548* RNAi. Knockdown of these genes in the JHA-treated workers (7th instar larvae) resulted in the decrease of developmental stages (just a single molt) and activation of soldier-specific morphogenesis with developed mandibles and well-tanned cuticle formations (Fig 2A). The Sox11 gene belongs to SOX (Sry-related HMG box) gene family, which has multiple functionalities including embryogenesis, sex determination, and neurogenesis both in vertebrates and invertebrates [19,20]. Although the function of *Sox11* is unclear in insects, it is involved in the regulation of embryonic development in mammals [21,22], probably through its relationship with TGF*β* signaling [21]. *Znev_01548* was a function-unknown gene, but interestingly it contained TGF*β* propeptide domain, functioned as configuration of LAP (latency-associated peptide) region in the TGF*β* ligand precursor protein to form homodimer with TGF*β* binding protein in mammals [23]. TGF*β* signaling is well known as a universal gene network of many biological functions in metazoans (reviewed in [24]). Importantly, it is involved in the regulation of JH biosynthesis in flies and crickets [25,16], and the activation of a 20E receptor gene (*EcR*) in crickets [17]. Present results are not inconsistent with these evidences. Namely, both *ZnSox11* and *Znev_01548* expression increased after JHA treatment, and was decreased by the knockdown of JH receptor gene, *Met* (Fig 2D, S6 Table). Moreover, knockdown of both genes inhibited the expression of the 20E signaling gene *E75* (also *EcR* in the case of *Znev_01548* RNAi) (Fig 3, S6 Table). One possibility of the function of TGF*β* signaling during soldier differentiation is a mediation between JH (high JH titer levels) and 20E (EcR and/or 20E signaling activation). The high *EcR* expression in the head just after the presoldier molt was necessary for the molt into a soldier from a presoldier in *Z*. *nevadensis* [11]. Consequently, TGF*β* signaling may modulate the double molting processes via the regulation of *EcR* and/or 20E signaling gene expression changes during soldier differentiation (Fig 4). Because double knockdown of *ZnSox11* and *Znev_01548* did not enhance the soldier-like phenotype, there may be a rate-limiting process in TGF*β* signaling during soldier differentiation. Further expression and function analysis of other TGF*β* signaling members would be required to clarify this possibility. Moreover, expression levels of both *ZnSox11* and *Znev_01548* also fluctuated during the worker molt (Fig 2C, S6 Table). To know whether TGF*β* signaling has another role during the worker molt, RNAi analysis should be performed.

### Evolution of soldier differentiation system in termites

All termite species with the soldier caste possess the presoldier stage and the double molting system during soldier differentiation [26]. The presoldier is not engaged in any tasks in the colony and is regarded as solely an intermediate stage for the soldier caste. Therefore, the double molting system may be necessary to generate soldier-specific defensive morphology. However, the present RNAi experiments produced the soldier-like phenotypes with only a single molt from workers, which possibly could be engaged in defensive tasks like natural soldiers. There is a possibility that these individuals with incomplete phenotypes are the first acquired soldiers during the course of termite evolution. The acquisition of JH dependent 20E regulatory system mediated by the TGF*β* signaling might then allow presoldier morphogenesis and complete soldier differentiation via the double molting process. To follow this hypothesis, expression and function of other genes in TGF*β* signaling should be analyzed in detail, especially in the cockroach lineages which are closely related to termites.

### Conclusion

We performed the RNA-seq analysis in the head during caste differentiation under natural conditions in *Z*. *nevadensis*. Two important genes related to TGF*β* signaling were detected as highly expressed genes before the presoldier molt. Functional analyses demonstrated that these genes were involved in the formation of presoldier-specific phenotypes and the activation of the next soldier molt. Based on the gene expression analyses, TGF*β* signaling may be involved in the mediation between JH and 20E signalings during termite soldier differentiation. Further analyses will elucidate the role of TGF*β* signaling for the double molting system required for soldier differentiation in modern termite species. The results of this study will provide new cutting edge data for the discussion of sterile caste evolution in eusocial insects.

## Materials and methods

### Insects

Several mature colonies of *Z*. *nevadensis* were collected from laurel forests in Hyogo Prefecture, Japan, in May 2013. All colonies were kept in plastic cases at approximately 25°C in constant darkness until the emergence of newly molted alates. In accordance with previous studies [12,27], 50 incipient colonies were founded by mating male and female alates in 40 mm plastic dishes, and these colonies were kept at approximately 25°C in constant darkness. According to the developmental schedule previously described in an incipient colony [9,12], individuals were collected at the following developmental stages during soldier differentiation (3rd instar—presoldier—soldier) and worker molt (3rd instar - 4th instar; Fig 1A); pre gut-purging (pGP), 0 days after gut-purging (GP0), 3 days after gut-purging (GP3), 0 days after molting (M0) and 3 days after molting (M3). For RNA interference (RNAi) experiments, 7th instars engaged in worker tasks in the mature colony were sampled from a colony collected in Hyogo Prefecture in May 2015. Individuals used for RNA extraction were immersed immediately in liquid nitrogen and stored at −80°C.

### RNA extraction

For RNA-sequencing analysis during each molt (worker, presoldier and soldier molts), total RNA was extracted from heads of three individuals using SV Total RNA isolation kit (Promega Madison, WI, USA). The amounts of RNA and DNA in each sample were quantified using a Qubit fluorometer (Life Technology, Eugene, OR, USA), and the quality of RNA was validated using an Agilent 2100 bioanalyzer (Agilent Technologies, Palo Alto, CA, USA). For real-time qPCR analysis to validate the results based on the RPKM (Reads Per Kilobase of exon model per Million mapped reads) values (see below), total RNA of each developmental stage was extracted from heads using ISOGEN (NipponGene, Tokyo, Japan). Biological triplicates derived from three different heads were prepared. For real-time qPCR analysis under the RNAi assay of candidate genes, total RNA of 7th instars was extracted from the whole body using ISOGEN. The extracted RNA was purified with RNase-free DNaseI (Takara, Shiga, Japan) for removing genomic DNA.

### RNA-seq library preparation

Total RNA (500 ng) was used for cDNA synthesis using a TrueSeq sample preparation kit (Illumina, San Diego, USA) according to the low throughput protocol. The amount of compound cDNA library was quantified using the qPCR method with a Library Quantification Kit—Illumina/Universal (KAPA Biosystems, Wilmington, USA). Each cDNA library (50 μL, 2 nM) was used for RNA-sequencing, which was performed by single-end 100 bp sequence with the next-generation sequencer Hiseq 2000 (Illumina). One library was prepared in each developmental stage and total 15 libraries were sequenced. All of the reads have been deposited in the DDBJ Sequence Read Archive (DRA) database under accession numbers DRA006300.

### Construction and comparison of RNA-seq data sets

The quality of all reads was checked by the FastQC program [28], and low quality reads were removed by SolexaQA software [29]. Adapter sequences were removed using the Cutadapt program [30]. Cleaned reads were mapped to genome sequence data (gene model OGSv2.2; [8]) using Tophat v2.0.8 software with Bowtie2 v2.1.0.0 [31]. MDS plots of all expressed genes (14,204 genes) were constructed based on the Jensen-Shannon distances by the cummeRbund package [32] using the RPKM values obtained. Expression rates were calculated by the cuffdiff command in Cufflinks software [32]. Highly expressed genes in the head before the presoldier molt were selected by comparison between the same developmental stages described above (pGP, GP0 and GP3) in two molts (worker and presoldier molts, presoldier and soldier molts). According to the expression patterns during molts, highly expressed genes in the head were clustered using the cummeRbund package [32]. Gene similarity searches were conducted using the blastx algorithm with blast+ package (version 2.3.0) [33] against the FlyBase (in case of no hits, non-redundant (nr) database) (performed on 15 May 2017) of GenBank in the NCBI server. We set an E-value threshold of ≤1e-4 and bit score threshold of ≥ 40 for BLAST hits.

### JHA treatment

According to the methods of Saiki et al. (2014) [34], filter paper was treated with 0 (for control) or 10 μg JHA (pyriproxyfen; Wako, Osaka, Japan) dissolved in 400 μl acetone and placed in a 90 mm petri dish with ten 7th instar individuals. All petri dishes were kept in an incubator at 25°C in constant darkness and checked for dead or newly molted individuals every 24 hours. For the expression analysis of candidate genes during the presoldier molt induced by the JHA treatment, individuals were sampled at the following 8 points; 10, 7, 4 and 1 day before the 1st (i.e. induced presoldier) molt, and 0, 3, 5 and 7 days after the 1st molt. Note that when the individuals were treated with JHA, the gut-purged individuals were observed approximately 7 days before the 1st molt (see results).

### RNA interference (RNAi) experiment

Double-strand RNA (dsRNA) of candidate genes was generated by the partial cDNA sequences amplified by the gene-specific primers (S3 Table) using T7 RNA polymerase with a MEGA script T7 transcription kit (Ambion, Austin, TX, USA). According to previous reports [9–11,34,35], *GFP* was selected as a control gene and dsRNA was generated using the GFP vector pQBI-polII (Wako, Osaka, Japan). Specific primers for the 13 target genes were designed from genome sequence data [8] using Primer3 plus software [36] (S3 Table). Specific primers of the JH receptor gene, *Methoprene tolerant* (*ZnMet*; *Znev_9570*) were designed according to the previous study (S3 Table; [9]). The amount of compound dsRNA was quantified by a NanoVue spectrophotometer (GE Healthcare Bio-Sciences, Uppsala, Sweden), and 500 ng of dsRNAs were injected into the side of the thorax of individuals using a Nanoliter 2000 microinjector (World Precision Instruments, Sarasota, FL, USA) at 24 hours after the JHA treatment (target genes except for *ZnSox11* and *Znev_01548*, n = 10; *ZnSox11*, n = 30; *Znev_01548*, n = 30; double knockdown of *ZnSox11* and *Znev_01548*, n = 10) or simultaneously with JHA treatment (*ZnMet*, n = 5). Phenotypes of the molted individuals were observed 7 days after the 1st molt. The 2nd (i.e. induced soldier) molting rates were measured at day 30 after the 1st molt. For the gene expression analysis under the RNAi treatment, the JHA-treated individuals were collected at 10, 7, 4 and 1 day before the 1st molt, and 0, 3, 5 and 7 days after the 1st molt.

### Quantification of RNAi effects

According to the methods of Ishikawa et al. (2012) [37], aggression levels of individuals were quantified by the frequency of biting against the wood ant *Formica japonica* as a hypothetical enemy. One RNAi-treated individual or natural soldier was placed in a 40 mm plastic dish with moistened filter paper, and a *F*. *japonica* placed on the end of a toothpick was presented for 3 min. The number of times that *F*. *japonica* was bitten were counted. These experiments were replicated 6–9 times using different individuals of both *Z*. *nevadensis* and *F*. *japonica*. To quantify the color of the head capsule, RNAi treated individuals and natural soldiers were preserved in the FAA solution (ethanol : formalin : acetic acid = 16 : 6 : 1) for 24 hours and stored in 70% ethanol. These fixed individuals were observed using a SZX10 stereomicroscope and 3CCD digital camera XD250-2D (Olympus, Tokyo, Japan). From the captured images of their head capsules, average color properties of the head capsule area were detected using the color picker of Adobe Photoshop CS software 7.0 (Adobe Systems Inc., San Jose, CA, USA). The color properties of each individual were evaluated as the average values of ten points randomly chosen for the HSB (hue angle, saturation, brightness) color model [c.f. 11, 39]. Kruskal-Wallis test and Steel-Dwass tests were performed for comparison among the RNAi-treated individuals and natural soldiers using the statistical software Mac Statistical Analysis ver. 2.0. (Esumi, Tokyo, Japan).

### Gene expression analysis using real-time qPCR

The cDNA was synthesized using a High Capacity cDNA Reverse Transcription Kit (Applied Biosystems). The expression level of each gene was quantified using THUNDERBIRD SYBR qPCR Mix (TOYOBO, Osaka, Japan) and a MiniOpticon Real-Time System detection system (Bio-Rad, Hercules, CA, USA). For the RNAi-treated 1st molting individuals (JHA-induced soldier-like individuals; see results), expression levels of the following 2 genes (*Troponin* and *Laccase2* (*Lac2*); hereafter, soldier characteristic genes) were analyzed to check whether these individuals were genetically similar with the natural soldiers. *Troponin*, muscle formation gene was highly expressed in soldiers of *Reticulitermes flavipes* [38]. In the present study, highly *Troponin* expression levels were observed in soldier heads after the molt (M3 library, RPKM value = 13479.6) compared with worker heads (M3 library, 130.9). *Lac2* was involved in the cuticular tanning during soldier differentiation and high expression levels were observed in heads of *R*. *speratus* [39] and *Z*. *nevadensis* soldiers [10]. Specific primers for qPCR of the following target genes, *ZnSox11* (*Znev_04641*), *Znev_01548*, *Troponin* (*ZnTro*; *Znev_06448*) and three ecdysone synthetic genes (*Neverland*: *ZnNvd*; *Znev_04416*, *Shroud*: *ZnShr*; *Znev_16529*, *Spook*: *ZnSpo*; *Znev_04417*) were designed from genome sequence data [8] using Primer3 plus software [36]. The qPCR primers of *Lac2* (*ZnLac2*; *Znev_17433*), *Ecdysone receptor* (*ZnEcR*; *Znev_13925*) and *Ecdysone-inducible protein E75* (*ZnE75*; *Znev_11406*) were previously described [10,11]. The following six house-keeping genes were used as endogenous controls for constitutive expression: *EF1-alfa* (DDBJ/EMBL/GenBank Accession No. AB915828), *beta-actin* (No. AB915826), *NADH-dh* (No. AB936819) and three ribosomal protein genes (*RS49*: GeneID: KDR21989, *RPS18*: KDR22651 and *RPL13a*: KDR22610) [8,10]. The most appropriate gene was evaluated using GeNorm [40] and NormFinder [41], and *EF1-alfa* was selected in all real-time qPCR analyses performed in this study (S4 Table). Relative expression levels of the target genes were calculated by adopting the standard curve method. Expression levels were calculated using biological triplicates. Prior to the use of ANOVA, we performed the Browne-Forsythe test on the variance equality. Post-hoc tests among each developmental stage were conducted using Scheffe’s F test. These statistic analyses were performed using the statistical software Mac Statistical Analysis ver. 2.0.

## Supporting information

S1 Fig Color property of head capsule of RNAi treated individuals and natural soldiers. The values of each color property (mean ± S.E., n = 5–8) observed from head capsules. Different letters over the bars indicate significant differences in each category (Kruskal-Wallis test: hue angle; P = 1.62E-05, saturation; P = 6.18E-05, brightness; P = 9.06E-05, Steel-Dwass test: P < 0.05). Numbers of individuals examined are shown in parentheses.
(TIFF)

S2 Fig cDNA sequences and deduced amino acid sequences of *Znev_01548*. The nucleotides in white letters indicate TGF*β* (Transforming growth factor beta) propeptide domain (E-value = 1.71E-04).
(TIF)

S3 Fig Gene expression patterns of ecdysone synthetic genes in the whole body during the presoldier molt induced by JHA treatment under the RNAi treatment of *ZnSox11* and *Znev_01548*. Gray, red and blue lines indicate the results under the *GFP*, *ZnSox11* and *Znev_01548* RNAi treatments, respectively. Relative expression levels (mean ± S.E., biological triplicates) were calibrated by the expression level of *GFP* dsRNA injected workers (-10) as 1.0. The statistical results of two-way ANOVA are described in each box (*P < 0.05, **P < 0.01). The data is consistent with the use of parametric statistics by the Browne-Forsythe test (*ZnNvd*: P = 7.32E-01 (*GFP*), 6.77E-01 (*ZnSox11* RNAi), 4.84E-01 (*Znev_01548* RNAi); *ZnShr*: P = 5.04E-01 (*GFP*), 7.02E-01 (*ZnSox11* RNAi), 6.96E-01 (*Znev_01548* RNAi); *ZnSpo*: P = 3.77E-01 (*GFP*), 7.21E-01 (*ZnSox11* RNAi), 5.86E-01 (*Znev_01548* RNAi)) prior to the use of the ANOVAs.
(TIF)

S1 Table RNA-seq data of all genes observed in this study. (XLSX)

S2 Table List of genes with highly expression levels in the head before the presoldier molt. Dmel gene ID and gene name indicate the FlyBase ID and name with E-value, respectively. In case of no hits with FlyBase, top hit with the non-redundant (nr) database of GenBank in the NCBI server is described in the line for gene name. Circles in the leftmost line (RNAi) indicate the target genes used for the function analysis (total 13). Astelisks in Gene ID indicate the transcription factors. See the text for more details.
(PDF)

S3 Table Phenotypes of JHA-treated workers after RNAi of 13 target genes. In the rightmost line (phenotype), 'normal' indicates the usual phenotype of induced presoldier, and 'lethal' means that most individuals (80–100%) died before the molt.
(PDF)

S4 Table Raw data for aggression level in Fig 2B. Data represents the number of times that *Formica japonica* was bitten for 3 min.
(XLSX)

S5 Table Raw data for color quantification in S1 Fig. Data represents the HSB color property of the head capsule area.
(XLSX)

S6 Table qRT-PCR data for Figs 2C, 2D, 3 and S3. Data represents relative expression levels calculated from standard curve in each gene.
(XLSX)

S7 Table Primer sequences used in this study. (PDF)

S8 Table Ranking and stability values of reference genes using GeNorm and NormFinder. (PDF)
