# Supplementary figures and images for: TGFβ signaling related genes are involved in hormonal mediation during termite soldier differentiation
Source: PLoS Genet. 2018 Apr 11;14(4):e1007338. doi: 10.1371/journal.pgen.1007338 (PMC5912798; doi:10.1371/journal.pgen.1007338)

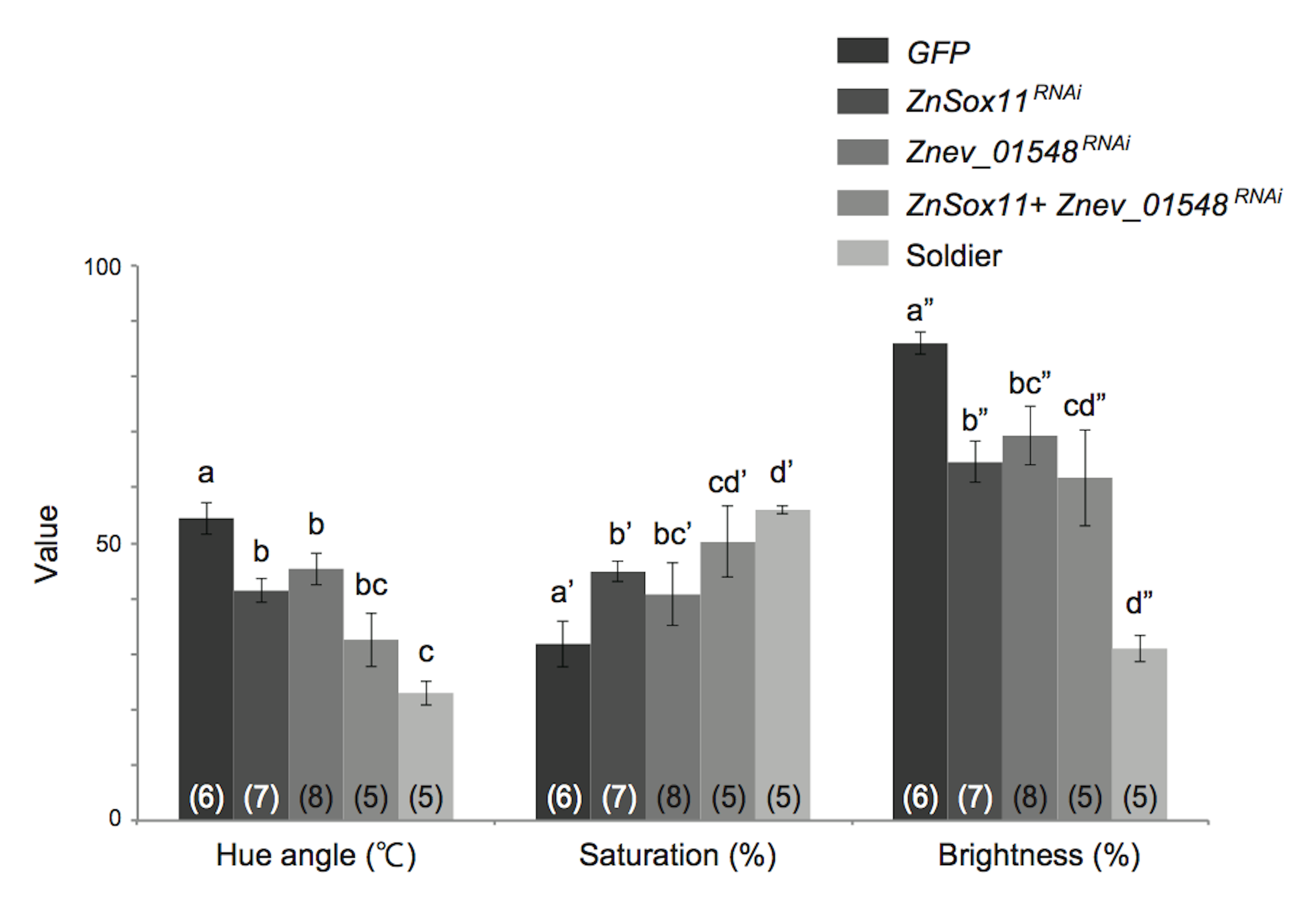

Supplement: S1 Fig — The values of each color property (mean ± S.E., n = 5–8) observed from head capsules. Different letters over the bars indicate significant differences in each category (Kruskal-Wallis test: hue angle; P = 1.62E-05, saturation; P = 6.18E-05, brightness; P = 9.06E-05, Steel-Dwass test: P < 0.05). Numbers of individuals examined are shown in parentheses. (TIFF) [file pgen.1007338.s001.tiff]

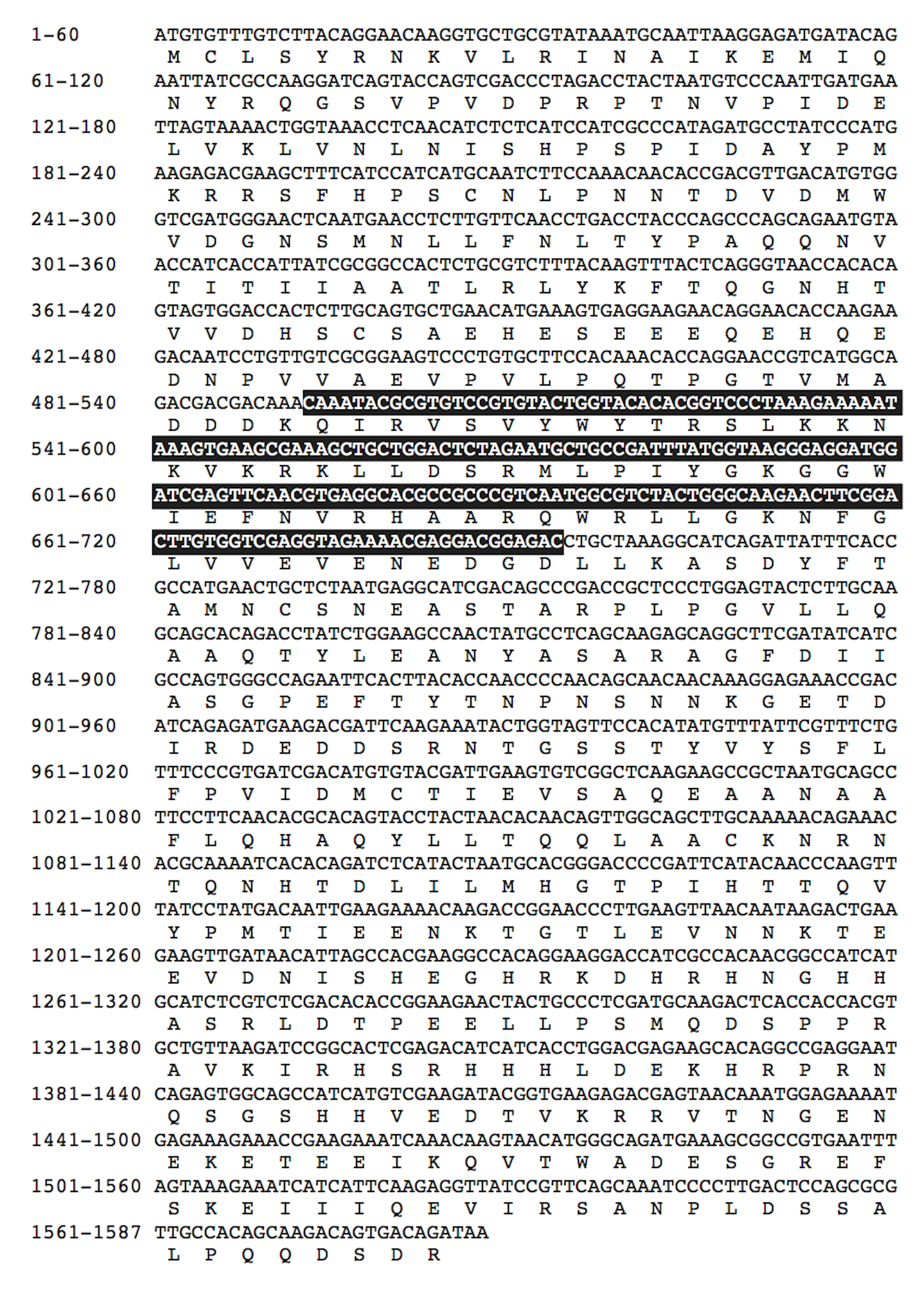

Supplement: S2 Fig — The nucleotides in white letters indicate TGFβ (Transforming growth factor beta) propeptide domain (E-value = 1.71E-04). (TIF) [file pgen.1007338.s002.tif]

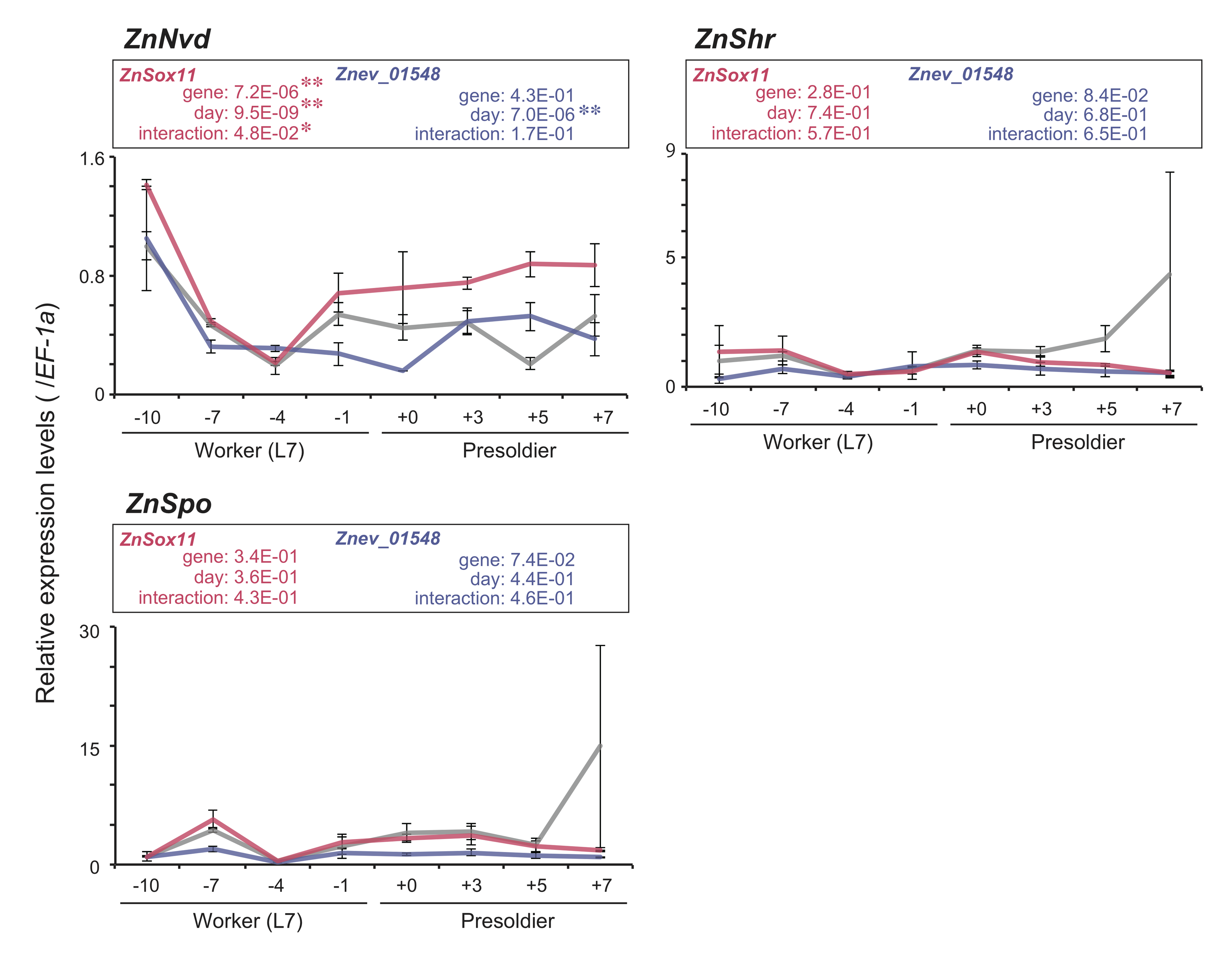

Supplement: S3 Fig — Gray, red and blue lines indicate the results under the GFP, ZnSox11 and Znev_01548 RNAi treatments, respectively. Relative expression levels (mean ± S.E., biological triplicates) were calibrated by the expression level of GFP dsRNA injected workers (-10) as 1.0. The statistical results of two-way ANOVA are described in each box (*P < 0.05, **P < 0.01). The data is consistent with the use of parametric statistics by the Browne-Forsythe test (ZnNvd: P = 7.32E-01 (GFP), 6.77E-01 (ZnSox11 RNAi), 4.84E-01 (Znev_01548 RNAi); ZnShr: P = 5.04E-01 (GFP), 7.02E-01 (ZnSox11 RNAi), 6.96E-01 (Znev_01548 RNAi); ZnSpo: P = 3.77E-01 (GFP), 7.21E-01 (ZnSox11 RNAi), 5.86E-01 (Znev_01548 RNAi)) prior to the use of the ANOVAs. (TIF) [file pgen.1007338.s003.tif]
