# Supplementary material for: TGFβ signaling related genes are involved in hormonal mediation during termite soldier differentiation
Source: PLoS Genet. 2018 Apr 11;14(4):e1007338. doi: 10.1371/journal.pgen.1007338 (PMC5912798; doi:10.1371/journal.pgen.1007338)
Supplement: S2 Table — Dmel gene ID and gene name indicate the FlyBase ID and name with E-value, respectively. In case of no hits with FlyBase, top hit with the non-redundant (nr) database of GenBank in the NCBI server is described in the line for gene name. Circles in the leftmost line (RNAi) indicate the target genes used for the function analysis (total 13). Astelisks in Gene ID indicate the transcription factors. See the text for more details. (PDF) [file pgen.1007338.s005.pdf]

S2 Table

RNAi	Gene ID	bp	Dmel gene ID	Dmel gene name (E-value)	top hit gene (blastx: nr)
	Znev_00050	1430	FBgn0051719	RluA-1 (1.85e-09)	uncharacterized protein C18B11.02c-like isoform X5 [*Zootermopsis nevadensis*]
	Znev_00105	156			no hit
○	Znev_00113	687			uncharacterized protein LOC110826764 [*Zootermopsis nevadensis*]
	Znev_00680	1635	FBgn0262599	CG43129 (2.92e-121)	protein msta [*Zootermopsis nevadensis*]
	Znev_01131	894	FBgn0020269	M-spondin (6.02e-50)	Spondin-2 [*Zootermopsis nevadensis*]
	Znev_01403	3414	FBgn0250907	Chitinase 10 (0)	probable chitinase 10 [*Zootermopsis nevadensis*]
○	Znev_01548	1587			hypothetical protein L798_14665 [*Zootermopsis nevadensis*] (0)
	Znev_02940	4083	FBgn0039527	CG5639 (7.57e-167)	balbiani ring protein 3-like [*Zootermopsis nevadensis*]
	Znev_03089	543			ADP-sugar pyrophosphatase [*Zootermopsis nevadensis*]
	Znev_03209	3966	FBgn0035410	CG14964 (1.18e-148)	Titin [*Zootermopsis nevadensis*]
	Znev_03214	825	FBgn0035308	CG15822 (1.69e-03)	LOW QUALITY PROTEIN: titin [*Zootermopsis nevadensis*]
○	Znev_03428	771	FBgn0250839	CG2016 (2.82e-74)	protein takeout isoform X1 [*Zootermopsis nevadensis*]
	Znev_03569	1152	FBgn0053978	CG33978 (9.94e-06)	mucin-17 [*Zootermopsis nevadensis*]
	Znev_04417	1506	FBgn0086917	spook (5.84e-137)	cytochrome P450 307a1-like [*Zootermopsis nevadensis*]
○	*Znev_04641	1137	FBgn0005612	Sox box protein 14 (5.14e-31)	transcription factor SOX-11-like isoform X3 [*Zootermopsis nevadensis*]
	Znev_05398	1638	FBgn0030304	Cyp4g15 (0)	cytochrome P450 4g15 [*Zootermopsis nevadensis*]
	Znev_05448	1737	FBgn0036282	Smyd4-2 (1.83e-113)	SET and MYND domain-containing protein 4 isoform X1 [*Zootermopsis nevadensis*]
	Znev_05559	654	FBgn0031918	CG6055 (2.06e-117)	uncharacterized protein LOC110828171 [*Zootermopsis nevadensis*]
○	*Znev_05644	1863	FBgn0038286	CG6966 (0)	Fem-1-like protein, partial [*Zootermopsis nevadensis*]
○	Znev_05682	1002			uncharacterized protein LOC110835378 isoform X1 [*Zootermopsis nevadensis*]
	Znev_05770	780	FBgn0030332	CG9360 (7.10e-57)	farnesol dehydrogenase-like [*Zootermopsis nevadensis*]
	Znev_05812	567	FBgn0035686	Cuticular protein 65Az (3.54e-16)	Endocuticle structural glycoprotein SgAbd-9, partial [*Zootermopsis nevadensis*]
	Znev_06341	669	FBgn0050418	nord (7.53e-14)	Fibronectin type-III domain-containing protein C4orf31-like protein [*Zootermopsis nevadensis*]
	Znev_06448	462	FBgn0013348	Troponin C at 41C (3.32e-55)	troponin C-like isoform X1 [*Zootermopsis nevadensis*]
	Znev_06647	1332	FBgn0041585	olf186-F (2.01e-61)	uncharacterized protein LOC110829337 isoform X1 [*Zootermopsis nevadensis*]
	Znev_08413	606	FBgn0029838	CG4666 (3.02e-64)	protein THEM6-like [*Zootermopsis nevadensis*]
	Znev_09452	1185	FBgn0035056	spatzle 6 (8.16e-112)	hypothetical protein L798_05509, partial [*Zootermopsis nevadensis*]
	Znev_09539	2391	FBgn0039601	CG1523 (5.23e-93)	WD repeat-containing protein 32-like protein [*Zootermopsis nevadensis*]
○	Znev_10002	1686			hypothetical protein L798_03836 [*Zootermopsis nevadensis*]
	Znev_10107	1899	FBgn0034417	CG15117 (0)	beta-glucuronidase [*Zootermopsis nevadensis*]
○	Znev_10647	2841			hypothetical protein L798_03663 [*Zootermopsis nevadensis*]
	Znev_10997	2154	FBgn0039897	CG1674 (5.25e-20)	flocculation protein FLO11 isoform X2 [*Zootermopsis nevadensis*]
○	*Znev_11299	1791	FBgn0003028	ovo (4.30e-81)	protein ovo-like [*Zootermopsis nevadensis*]
	Znev_11433	1419	FBgn0037684	CG8129 (8.36e-146)	uncharacterized protein LOC110832432 isoform X2 [*Zootermopsis nevadensis*]
	Znev_11795	2697	FBgn0033095	CG3409 (5.85e-64)	uncharacterized protein LOC110839016 [*Zootermopsis nevadensis*]
	Znev_11891	987	FBgn0029994	CG2254 (1.40e-34)	epidermal retinol dehydrogenase 2-like [*Zootermopsis nevadensis*]
○	Znev_12514	333			hypothetical protein L798_13869 [*Zootermopsis nevadensis*]
	Znev_12919	3582	FBgn0039415	CG6142 (1.42e-153)	Glucose dehydrogenase [acceptor], partial [*Zootermopsis nevadensis*]
○	Znev_12943	342			hypothetical protein L798_11596 [*Zootermopsis nevadensis*]
	Znev_13044	480	FBgn0010423	Troponin C at 47D (1.96e-61)	troponin C-like [*Zootermopsis nevadensis*]
	Znev_14541	738	FBgn0034267	CG4984 (4.08e-43)	transmembrane protein 114 [*Zootermopsis nevadensis*]
	Znev_14677	1578	FBgn0033696	Cyp6g2 (8.72e-107)	cytochrome P450 6k1-like [*Zootermopsis nevadensis*]
	Znev_14747	888	FBgn0033807	aquaporin (9.14e-75)	aquaporin-11 [*Zootermopsis nevadensis*]
	Znev_15091	735			glycine-rich protein 5-like isoform X1 [*Zootermopsis nevadensis*]
	Znev_15416	696	FBgn0050045	Cuticular protein 49Aa (7.79e-25)	Endocuticle structural glycoprotein SgAbd-2, partial [*Zootermopsis nevadensis*]
	Znev_15605	1251			probable GPI-anchored adhesin-like protein PGA55 isoform X1 [*Zootermopsis nevadensis*]
○	Znev_15631	306			hypothetical protein L798_09057, partial [*Zootermopsis nevadensis*]
○	Znev_16430	354			hypothetical protein L798_05095 [*Zootermopsis nevadensis*]
	Znev_16954	1386	FBgn0030576	CG15890 (1.63e-162)	solute carrier family 46 member 3-like isoform X1 [*Zootermopsis nevadensis*]
	Znev_17430	4302	FBgn0035798	faulty attraction (1.92e-136)	fibrillin-1-like [*Zootermopsis nevadensis*]
	Znev_17630	5346	FBgn0010482	lethal (2) 01289 (0)	hypothetical protein L798_08259, partial [*Zootermopsis nevadensis*]
	Znev_18246	1299	FBgn0011817	nemo (0)	LOW QUALITY PROTEIN: serine/threonine-protein kinase NLK [*Zootermopsis nevadensis*]
	Znev_18583	1149	FBgn0033644	Trehalose transporter 1-2 (9.22e-137)	facilitated trehalose transporter Tret1-like isoform X1 [*Zootermopsis nevadensis*]
	Znev_19022	1430	FBgn0035468	Gustatory receptor 63a (2.00e-44)	gustatory and odorant receptor 24-like [*Zootermopsis nevadensis*]
