# Supplementary material for: TGFβ signaling related genes are involved in hormonal mediation during termite soldier differentiation
Source: PLoS Genet. 2018 Apr 11;14(4):e1007338. doi: 10.1371/journal.pgen.1007338 (PMC5912798; doi:10.1371/journal.pgen.1007338)
Supplement: S3 Table — In the rightmost line (phenotype), 'normal' indicates the usual phenotype of induced presoldier, and 'lethal' means that most individuals (80–100%) died before the molt. (PDF) [file pgen.1007338.s006.pdf]

S3 Table

Gene ID	Zn gene name	molting rate	mortarity	phenotype
Znev_00113	uncharacterized protein LOC110826764	60% (6/10)	0% (0/10)	normal
Znev_01548	hypothetical protein L798_14665	90% (27/30)	0% (0/30)	soldier like
Znev_03428	protein takeout isoform X1	80% (8/10)	0% (0/10)	normal
Znev_04641	transcription factor SOX-11-like isoform X3	83% (25/30)	0% (0/30)	soldier like
Znev_05644	Fem-1-like protein	0% (0/10)	80% (8/10)	lethal
Znev_05682	uncharacterized protein LOC110835378 isoform X1	60% (6/10)	0% (0/10)	normal
Znev_10002	hypothetical protein L798_03836	70% (7/10)	20% (2/10)	normal
Znev_10647	hypothetical protein L798_03663	90% (9/10)	10% (1/10)	normal
Znev_11299	protein ovo-like	10% (1/10)	70% (7/10)	normal
Znev_12514	hypothetical protein L798_13869	70% (7/10)	10% (1/10)	normal
Znev_12943	hypothetical protein L798_11596	80% (8/10)	10% (1/10)	normal
Znev_15631	hypothetical protein L798_09057	0% (0/10)	100% (10/10)	lethal
Znev_16430	hypothetical protein L798_05095	0% (0/10)	100% (10/10)	lethal
