# Supplementary material for: TGFβ signaling related genes are involved in hormonal mediation during termite soldier differentiation
Source: PLoS Genet. 2018 Apr 11;14(4):e1007338. doi: 10.1371/journal.pgen.1007338 (PMC5912798; doi:10.1371/journal.pgen.1007338)
Supplement: S7 Table — (PDF) [file pgen.1007338.s010.pdf]

S7 Table

Gene	Forward	Reverse
ZnSox11 (dsRNA)	T7+ACCACTCCCCAAAGTGTCTG	T7+AAGGTGAATGTGTGGCTTCC
Znev_01548 (dsRNA)	T7+GAAGCCAACTATGCCTCAGC	T7+CTCGAGTGCCGGATCTTAAC
Znev_00113 (dsRNA)	T7+CAGGGACATTCGGAAAGAAA	T7+TCGTATTCCTCGTTGGAAGG
ZnCG2016 (dsRNA)	T7+CAGAGGTATTCCGGAACTGG	T7+CGAAGTTCCTCGTCTTCTGG
ZnCG6966 (dsRNA)	T7+AGGACATTTGCCAGTGGTTC	T7+GCTCCAAGAAGTTCCAGTGC
Znev_05682 (dsRNA)	T7+ATGGTAACGGATTCCACCAA	T7+TACCCCGAAGGTACGAACTG
Znev_10002 (dsRNA)	T7+CACCTTCCGATACAGCCATT	T7+ACGGCGACTTGGAATATGAC
Znev_10647 (dsRNA)	T7+TTCATCTGGTGCTGCTCAAC	T7+GTTGCAGCCGTGGTTTTAAT
Znovo (dsRNA)	T7+TCAACGTTCTGCAACAGAGG	T7+TGCGTACTGATGCTGGACTC
Znev_12514 (dsRNA)	T7+CATTATGGCGGTTTCAGGTT	T7+CCGTTGATAGACGATGCAGTT
Znev_12943 (dsRNA)	T7+AGCGATCAGCTGTTTCTGGT	T7+GACCGCTTCATCCCCTAGA
Znev_15631 (dsRNA)	T7+AAGCCAGTTATCCCTGTGGA	T7+GATCCCCATCGAGGACAGT
Znev_16430 (dsRNA)	T7+GATCGGGAATCTCACCACAT	T7+TGATATTTCCAAGCCCGTTC
ZnMet (dsRNA)	T7+GAGGATGATCAGGGTGGAGA	T7+TCGAAGCTGTTCATCTGGTG
ZnSox11 (qPCR)	GACGCAAGATATGCGAACAA	AAGGGCTGTCTTTCCTCCTC
Zn_01548 (qPCR)	TCCATCATGCAATCTTCCAA	GCTGGGTAGGTCAGGTTGAA
ZnMet (qPCR)	ATCACGATGTGCTGGACAAG	GTGATGATGAACTGGAGGTGTC
ZnTro	GAGGCCATGCAACAAGAACT	TTCAGAATCTCACGCAGCAC
ZnLac2	TCGACTTCGGACAGAGAGGT	CATATCTTCGGAGGCTCGTC
ZnEcR	CCACAGAGCAGTCTGAATGG	GAGACACAACTCCTCCTGCTG
ZnE75	CCGCAGAACTTGAAGATGAG	AGCATGGGTTCCACTTTGTC
ZnNvd	CGTCGTTCTACCGATGGATT	CACTTTCTCCTGCCTTTTCG
ZnShr	CTGGGAAGGAAGGAGGATTC	CAGTTTATACGCGTGCAAGG
ZnSpo	AAAATCGTGGCCAATAATCG	CACGACGTCACCATAACGAC
Znb-actin	AGCGGGAAATCGTCCGTGAC	CAATGGTGATGACCTGCCCAT
ZnEF-1a	GCATGCACTGTTGGCTTTTA	TTCCTCAAATCGGGTTTCAG
ZnNADH-dh	CGGCAAGGAAGCAAATAAAG	TTGGGTTGGGGGTATCAGC
ZnRS49	CATGCTTCCTACTGGCTTCC	AATTTCGGCACAGAATTTGC
ZnRPS18	CTCCGTGAAGACCTGGAGAG	CGTCTTCGTGTGTTGTCCAC
ZnRPL13a	CACTTCAGAGCACCAAGCAA	ACGTTTCAATGCTGCCTTTC
T7 promorter	TAATACGACTCACTATAGGG	
