# Supplementary material for: TGFβ signaling related genes are involved in hormonal mediation during termite soldier differentiation
Source: PLoS Genet. 2018 Apr 11;14(4):e1007338. doi: 10.1371/journal.pgen.1007338 (PMC5912798; doi:10.1371/journal.pgen.1007338)
Supplement: S8 Table — (PDF) [file pgen.1007338.s011.pdf]

S8 Table

| | GeNorm (stavility value) | NormFinder (stavility value) |
|---|---|---|
| Fig.2C | | |
| | *EF1-alfa* (0.732) | *EF1-alfa* (0.079) |
| | *RPL13a* (0.763) | *RS49* (0.226) |
| | *RS49* (0.799) | *RPL13a* (0.384) |
| | *RPS18* (0.884) | *NADH-dh* (0.494) |
| | *NADH-dh* (1.229) | *RPS18* (0.575) |
| | *beta-actin* (1.53) | *beta-actin* (0.914) |
| Fig.2D | | |
| | *EF1-alfa* (0.318) | *EF1-alfa* (0.096) |
| | *RPS18* (0.318) | *RPS18* (0.111) |
| | *NADH-dh* (0.362) | *NADH-dh* (0.137) |
| | *RS49* (0.363) | *RS49* (0.155) |
| | *RPL13a* (0.395) | *RPL13a* (0.229) |
| | *beta-actin* (0.568) | *beta-actin* (0.369) |
| Fig.3, S3 | | |
| | *EF1-alfa* (0.697) | *EF1-alfa* (0.147) |
| | *NADH-dh* (0.704) | *NADH-dh* (0.147) |
| | *RPS18* (0.742) | *RPS18* (0.286) |
| | *RS49* (0.809) | *RS49* (0.383) |
| | *RPL13a* (1.155) | *RPL13a* (0.696) |
| | *beta-actin* (1.272) | *beta-actin* (0.811) |
